# DTI Parameter Optimisation for Acquisition at 1.5T: SNR Analysis and Clinical Application

**DOI:** 10.1155/2010/254032

**Published:** 2010-01-05

**Authors:** M. Laganà, M. Rovaris, A. Ceccarelli, C. Venturelli, S. Marini, G. Baselli

**Affiliations:** ^1^Polo Tecnologico, Fondazione Don Gnocchi ONLUS, IRCCS S. Maria Nascente, 20148 Milano, Italy; ^2^Department of Bioengineering, Politecnico di Milano, 20133 Milan, Italy; ^3^U.O. Sclerosi Multipla, Fondazione Don Gnocchi ONLUS, IRCCS S. Maria Nascente, 20148 Milano, Italy

## Abstract

*Background*. Magnetic Resonance (MR) diffusion tensor imaging (DTI) is able to quantify in vivo tissue microstructure properties and to detect disease related pathology of the central nervous system. Nevertheless, DTI is limited by low spatial resolution associated with its low signal-to-noise-ratio (SNR). *Aim*. The aim is to select a DTI sequence for brain clinical studies, optimizing SNR and resolution. *Methods and Results*. We applied 6 methods for SNR computation in 26 DTI sequences with different parameters using 4 healthy volunteers (HV). We choosed two DTI sequences for their high SNR, they differed by voxel size and b-value. Subsequently, the two selected sequences were acquired from 30 multiple sclerosis (MS) patients with different disability and lesion load and 18 age matched HV. We observed high concordance between mean diffusivity (MD) and fractional anysotropy (FA), nonetheless the DTI sequence with smaller voxel size displayed a better correlation with disease progression, despite a slightly lower SNR. The reliability of corpus callosum (CC) fiber tracking with the chosen DTI sequences was also tested. *Conclusions*. The sensitivity of DTI-derived indices to MS-related tissue abnormalities indicates that the optimized sequence may be a powerful tool in studies aimed at monitoring the disease course and severity.

## 1. Introduction

Magnetic Resonance (MR) diffusion tensor imaging (DTI) allows in vivo examination of the tissue microstructure, obtained by exploiting the properties of water diffusion. The DT computed for each voxel allowed us to calculate the magnitude of water diffusion, reflected by the mean diffusivity (MD) and the degree of anisotropy, which is a measure of tissue organization, expressed as an a-dimensional index, such as fractional anisotropy (FA) [1]. The pathological elements of multiple sclerosis (MS) have the potential to alter the permeability or geometry of structural barriers to water diffusion in the brain. Consistent with this, several in vivo DTI studies have reported increased MD and decreased FA values in T2-visible lesions, normal-appearing (NA) white matter (WM), and grey matter (GM) from patients with MS [2]. Combined with fibre tractography techniques, DTI reveals WM fibers characteristics and connectivity in the brain noninvasively. In MS, tractographic reconstruction has to deal with a general FA reduction in normal appearing white matter (NAWM) and a high FA reduction in lesions with high structural loss [2–5].

The best acquisition and postprocessing strategies for DTI sequences in the disease, especially in MS, are still a matter of debate [2, 6, 7].

The Signal-to-noise ratio (SNR) of an image is a fundamental measure of MRI-scanner hardware and software performances, because it provides a quantitative evaluation and comparison among signal and noise levels of different imaging and reconstruction methods, sequence parameters, radio frequency coils, gradient amplitudes, and slew rates. Since DT is reconstructed through evaluations of loss of signal in diffusion-weighted images in comparison with reference *b* = 0 s/mm^2^ images, this technique is vulnerable to poor SNR values: the background noise level close to the low diffusion weighted signal would overestimate the signal itself and consequently underestimate the magnitude of diffusion. The SNR of the *b* = 0 s/mm^2^ images should be at least 20 to obtain unbiased DTI-derived measures. Many methods for SNR evaluation in MR images are available and they differ for the estimation of the noise variance. They are commonly subdivided into two classes: single magnitude image methods derive the noise from a large, uniform background region [8, 9]; pair of images methods are based on two acquisitions of the same image [10–13]. The latter methods estimate the noise in the image obtained as the difference of the two acquired images, in a region positioned in the background or in the object of examination. These methods were not used for diffusion weighted evaluations, but only for conventional (T1,T2) imaging and validated on phantoms.

Against this background, the first aim of this study is the optimization of DTI sequence parameters, in order to produce images with high SNR, with a short acquisition time and a voxel size appropriate for tractography. The SNR was computed in brain images obtained with different DTI sequence parameters.

The second aim is the choice of the DTI sequence giving the best differentiation between HV and patients with MS.

The third aim is to ascertain whether these sequences enable us to track the corpus callosum (CC) fibers in MS patients [14–16]. 

A preliminary validation of the method will be shown on a group of MS patients with varying progression levels of the disease compared with an age-matched group of HV.

## 2. Material and Methods

### 2.1. Subjects

To obtain the optimization of SNR parameters, we performed a preliminary analysis on 4 HV (male/female = 2/2), mean age (range) = 44.75 (28–61) years).

To obtain the DTI sequence with the best differentiation between HV and MS patients we acquired 18 HV (male/female = 10/8, mean age (range) = 43.11 (24–50) years) and 30 MS patients (male/female = 8/22, mean age (range) = 45.03 (26–68) years, median EDSS (range) = 5.0 (2–8), median (range) disease duration = 13.5 (2–34) years), of whom 13 with relapsing-remitting (RR) MS and 17 with secondary progressive (SP) MS.

### 2.2. MRI Acquisition

MR scans were performed using a 1.5 T Siemens Magnetom Avanto scanner (Erlangen, Germany) in the Radiology Department of Fondazione Don Gnocchi ONLUS, IRCCS S. Maria Nascente, Milano (Italy). 

Twenty-six DTI sequences with different parameters were tested on 4 HV for the preliminary analysis. Changed parameters were pixel size (from 1.87 to 2.5 mm^2^), slice thickness (from 1.9 to 2.8 mm), *b*-value (900 s/mm^2^, 1000 s/mm^2^, 1500 s/mm^2^, 2000 s/mm^2^), echo time (TE) (from 83 to 110 ms), and repetition time (TR) (from 6500 ms to 7800 ms).

The following reference sequences were applied on all 48 subjects of the study:

- dual-echo turbo spin echo (TSE) (TR = 2650 ms, TE = 28/113 ms, echo train length (ETL) = 5; flip angle = 150; 50 interleaved, 2.5 mm-thick axial slices, matrix size = 256 × 256 and a field of view (FOV) = 250 mm);
- three-dimensional (3D) T1-weighted magnetisation-prepared rapid acquisition gradient echo (MP-RAGE) (TR = 1900 ms, TE = 3.37 ms, TI = 1100 ms, flip angle = 15°, 176 contiguous, axial slices with voxel size = 1 × 1 × 1 mm^3^, matrix size = 256 × 256, FOV = 256 mm, slab tick = 187.2 mm).

The following two DTI sequences were also applied, as a consequence of the previous screening on 4 HV:

- (DTI-A): pulsed-gradient spin-echo echo planar pulse sequence without SENSE (TR = 7000 ms, TE = 94 ms, 50 axial slices with 2.5 mm slice thickness, acquisition matrix size = 128 × 96; FOV = 320 × 240 mm) with diffusion gradients (*b*-value = 900 s/mm^2^) applied in 12 noncollinear directions;
- (DTI-B): pulsed-gradient spin-echo echo planar pulse sequence without SENSE (TR = 6500 ms, TE = 95 ms, 40 axial slices with 2.5 mm slice thickness, acquisition matrix size = 128 × 128; FOV = 240 × 240 mm) with diffusion gradients (*b*-value = 1000 s/mm^2^) applied in 12 noncollinear directions. Two acquisitions for each set of diffusion gradients were performed, in order to improve SNR. Acquisition time is compatible with clinical protocols: 3′09′′ for the first sequence (DTI-A) and 2′56′′ for the second (DTI-B).

The main differences between the first and the second DTI sequences were *b*-value (900 s/mm^2^ versus 1000 s/mm^2^), pixel size (2,5 mm × 2,5 mm versus 1,88 mm × 1,88 mm), and TR (7000 ms versus 6500 ms). 

DTI-B had 10 slices less than DTI-A; so it covered 25 mm less in the craniocaudal direction. Since our clinical aim is to analyze the microscopic changes of CC due to the MS pathology, we positioned DTI-B group of slices (slab) with the same centre and orientation of DTI-A slab, and then we moved it upward of 12,5 mm (25/2 mm) in the cranial direction. So, the two DTI had the last slice with the same position and orientation.

### 2.3. Methods for SNR Computation

All the 26 sequences were automatically analyzed with a home-made Matlab script, which computed SNR with six different methods for every slice of every volume (two *b*0 volumes, not diffusion-weighted, and twenty-four diffusion-weighted volumes) and plotted SNR-to-slice (Figure 2). 

**Figure 1:**
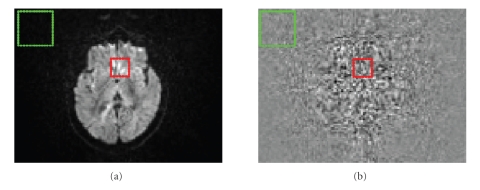
ROIs superimposed on 25th slice of the DTI-A 6th diffusion direction (a) and on image obtained by the difference of two acquisitions of the same image (b). The red ROI is for the evaluation of signal (for all the methods) and for the evaluation of noise standard deviation in methods 1 and in double image methods 2, 5; the green ROI is for the evaluation of noise in single image methods 3, 4, 6.

In all of the 6 methods, the signal (*S*) is evaluated as the 2D mean intensity in a region of interest (ROI) of 10 × 10 = 100 pixels with maximum uniform brain signal, automatically extracted for every slice (red ROI, Figure 1(a)). Instead, for the estimation of noise, single and multiple images methods were used. Even if the multiple images ones are relatively insensitive to structured noise such as ghosting, ringing, and direct current (DC) artifacts, a perfect geometrical alignment of the images and temporal steadiness of the imaging process are strict requirements. For this reason, corresponding volumes of the two subsequent acquisitions were previously coregistered with statistical parametric mapping (SPM)5 (http://www.fil.ion.ucl.ac.uk/spm/).

**Method 1—Single ROI for Signal and Noise, Single Image**

The noise was evaluated in the same ROI used for the S (see above). SNR is computed with (1) [17]:

(1) $$\text{SN}{\text{R}}_{1}=\frac{S}{\sigma },$$

where *σ* is the 2D standard deviation (SD) of pixel intensity in the ROI.

**Method 2—Single ROI for Signal and Noise, Difference of Images**

The noise was evaluated in the image obtained from the difference of two subsequent acquired images as the 2D SD of the intensities in the same ROI used for the signal S. Noise ROI must be positioned in tissue with sufficiently high SNR and not in the image background, because the noise within the ROI in the difference image is assumed to be Gaussian distributed.

SNR was then computed with (2) [17–21], where the factor $\sqrt{2}$ is due to the property of the addition of the variances when two images are added or subtracted:

(2) $$\text{SN}{\text{R}}_{2}=\sqrt{2}S/\sigma ,$$

where *σ* is the 2D SD of pixel intensity in the ROI.

**Method 3—Noise Estimated on Air (SD), Single Image**

The noise was estimated in a ROI of 20 × 20 = 400 pixels, extracted from background (air) (Figure 1(a)), paying attention to put it far from ghosting and filter artifacts, visible as an increased signal near image edges. Since MRI noise in the air follows Rayleigh distribution, the apparent SD of the noise underestimates the true SD by approximately 0.655. Therefore, the SNR was obtained by (3) [9, 20, 22] as

(3) $$\text{SN}{\text{R}}_{3}=\frac{S}{\text{SD}\left(\text{true}.\text{noise}\right)}=0.655\frac{S}{\text{SD}\left(\text{apparent}.\text{noise}\right)}.$$

**Method 4—Noise Estimated on Air (Mean Value), Single Image**

The standard deviation of noise was estimated from a ROI of 20 × 20 = 400 pixels, extracted from background (air). Since MR noise in the air follows Rayleigh distribution, the mean value of the signal in the second ROI (*μ*_air_) is equal to the SD of the noise, multiplied for the coefficient $\sqrt{\pi /2}$. So, SNR was computed with (4) [17, 20]:

(4) $$\text{SN}{\text{R}}_{4}=\sqrt{\frac{\pi }{2}}\cdot \frac{S}{\mu_{\text{air}}}.$$

**Method 5—Single ROI for Signal and Noise, Difference of Images**

This method was similar to the method 2. We considered two images (A and B) obtained from two subsequent acquisitions of the same slice. The signal was the mean value of the pixels in a ROI on the first image (A). Then, we considered a second ROI on the second image (B), located as the first ROI in the first image. The SD of the noise was evaluated in the same ROI position and computed as suggested by Ogura et al. [17] with (5):

(5) $$\sigma^{}=\sqrt{\tau_{\text{ROIA-ROIB}}^{2}+\tau_{\text{ROIB-ROIA}}^{2}+2\cdot \nu_{\text{ROIB-ROIA}}\cdot \nu_{\text{ROIA-ROIB}}},$$

where *τ* was the standard deviation and *ν* was the mean value of the pixel in an image obtained as the difference of image A minus image B (ROIA-ROIB) or vice versa.

**Method 6—Estimation of Noise Variance from the Background Histogram Mode, Single Image**

Since MRI noise in the air follows Rayleigh distribution, the noise variance can be estimated by searching for the magnitude (*m*) value at which the background histogram attains a maximum (*m*_max _air): noise SD was estimated as the mode of the Probability Density Function histogram [12, 23] in a background ROI of 20 × 20 = 400 pixels and the SNR was computed with (6):

(6) $$\text{SN}{\text{R}}_{6}=\frac{S}{\sigma_{\text{air}}}=\frac{S}{m_{\max}\;\left(\text{air}\right)}.$$

### 2.4. Postprocessing of Conventional Imaging

Lesions were segmented on proton-density(PD)-weighted images, using the corresponding T2-weighted images to increase confidence in lesion identification. Then, lesion volume (ml) was calculated and segmented lesions were used for masking DTI (see Section 2.5), using Jim software package (Jim 5.0, Xinapse System, Leicester, UK).

3D-T1 MP-RAGE images were automatically segmented to GM, WM and cerebrospinal fluid (CSF), using SPM5 (http://www.fil.ion.ucl.ac.uk/spm/) and maximum image in-homogeneity correction [24]. An home-made Matlab script was used to classify each pixel as GM, WM or CSF, dependent on which map had the greatest probability at that location: this produced mutually exclusive masks for each tissue.

### 2.5. Post Processing of Diffusion Tensor Imaging

DTI data were corrected for eddy-current distortion by FSL package, which registered the 12 diffusion-weighted volumes to the *b*0-volume, with a Mutual Information- (MI-) based nonlinear transformation. Then diffusion gradient directions were corrected for scanner settings (i.e., slice angulation, slice orientation, etc.) and diffusion tensor was determined for each voxel using the freely available Diffusion Toolkit software, version 0.4.2 (http://www.trackvis.org/) with linear least-squares fitting method [25]. The tensors were then diagonalized, obtaining eigenvectors, eigenvalues, MD, and FA maps.

ROIs of lesions individuated on T2-images were masked out from MD and FA maps, in order to estimate NAWM damage.

GM and WM mutual exclusive masks were superimposed to MD and FA maps, and the corresponding histograms were produced. The erosion of the first-line outer voxels from the mutual exclusive masks excluded the contribution of partial volume effect from the surrounding CSF to the observed GM and WM diffusivity changes and WM anisotropy changes. Average MD was computed for GM and NAWM. Average FA was derived only for the NAWM, since no preferential direction of water molecular motion is expected to occur in the GM, due to the absence of a microstructural anisotropic organization of this tissue compartment.

### 2.6. Fiber Tracking

The reliability of fiber tracking with the 2 DTI sequences was tested using Diffusion Toolkit v0.4.2 (http://www.trackvis.org/) and visualized by the freely available software TrackVis v0.4.2 (http://www.trackvis.org/). The brute force approach and deterministic streamline-based fiber tracking were used, with FA-map as masking image and angle termination of 35°. For track selection, the one-ROI approach was used: CC was identified and segmented in the three mid-sagittal adjacent slices of FA-map [26]. 

FA and MD histograms were derived for CC fiber tracts (CC-FA and CC-MD).

### 2.7. Statistical Analysis

A graphical display allowed to compare the six methods of SNR estimation and the quality of the sequences in terms of SNR.

We estimate the intraclass-correlation coefficients between the 2 DTI sequences used in the study, regarding the values of NAWM-FA, NAWM-MD, and GM-MD of all the 48 subjects (HV and MS patients). 

Spearman's correlation coefficient (SCC) was assessed to estimate the correlation between DTI-derived measures (NAWM-FA, NAWM-MD, GM-MD, CC-FA, and CC-MD) and the subjects' condition (HV, RRMS, SPMS).

## 3. Results

### 3.1. Analysis of SNR

**Figure 2:**
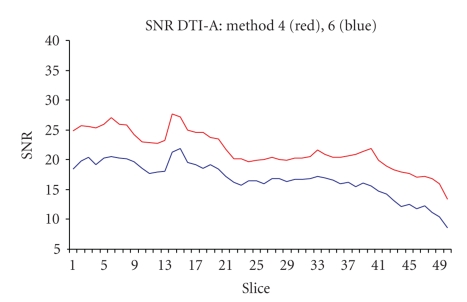
Comparison of DTI-A SNR obtained with method 4 and method 6. The mean SNR for *b* ≠ 0 s/mm^2^ images is plotted for every slice.

**Figure 3:**
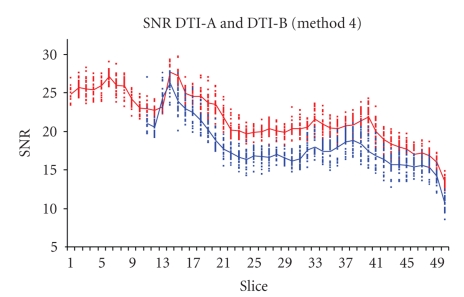
SNR computed with method 4 for images obtained with two repetitions of 12 DTI gradient directions. DTI-A (*b*-value = 900 s/mm^2^, 50 slices) (red) is compared with DTI-B (*b*-value = 1000 s/mm^2^, 40 slices) (blue). Note that DTI-B has been obtained with the last slice (*z* direction from feet to head) positioned as the last slice of DTI-A slice group.

**Figure 4:**
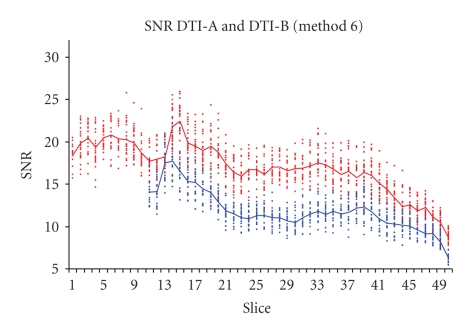
SNR computed with method 6 for images obtained with two repetitions of 12 DTI gradient directions. DTI-A (*b*-value = 900 s/mm^2^, 50 slices) (red) is compared with DTI-B (*b*-value = 1000 s/mm^2^, 40 slices) (blue). Note that DTI-B has been obtained with the last slice (*z* direction from feet to head) positioned as the last slice of DTI-A slice group.

As expected, the six SNR evaluation methods gave different absolute numerical values. Nevertheless, the changes through slices (Figure 2) and through different volumes were in good agreement, as the ranking of the performances of the different sequences (Figures 3, 4). 

SNRs were plotted for sequences ordered by ascending voxel size and with the same *b*-value, TE and TR: this kind of graphical representation showed clearly the increase of SNR with the increase of the voxel size. A similar representation was done for sequences with the same parameters but the *b*-value, giving the result of SNR decreasing with the increasing of the diffusion-sensitivity coefficient, in particular the SNR estimated on images obtained from sequences with *b*-value of 1500 s/mm^2^ was 20% less than the SNR of sequences with *b*-value of 1000 s/mm^2^. The same analysis confirmed that the minimum TE feasible for the MR-scanner had to be selected, as expected, since DTI is T2 weighted.

The sequence with the highest SNR by all methods was DTI-A, which is characterized by parameters in the range recommended by Pagani et al. [27] for multicentre MS trials.

Another sequence (DTI-B) was selected for the high SNR between those of pixel size of about 1 × 1 mm^2^. DTI-B SNR is lower than DTI-A SNR, less than 15%.

The SNR comparison of the two selected sequences is shown in Figures 3 and 4: only two SNR computational methods are shown (method 4 in Figure 3 and method 6 in Figure 4), but in both figures it is clear that DTI-A produces images with higher SNR, with near constant differences among slices.

### 3.2. Statistical Comparison of Microstructural Indices of Fiber Integrity, Derived from Two Sequences

The intraclass-correlation coefficients ranged from 0.91 to 0.99, showing high concordance of the parameters derived from DTI-A and DTI-B (Table 1).

The SCC showed that both DTI sequences separated HV from RRMS and SPMS patients, but that SCCs between DTI-B were higher than those between DTI-A (*P* < .01) and subjects' condition as shown in Table 2.

### 3.3. Fiber Tracking

**Figure 5:**
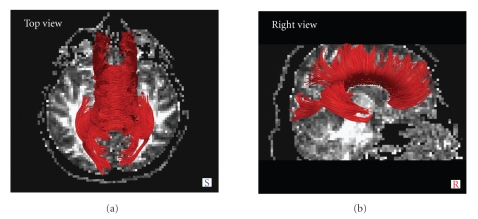
Top and right view of corpus callosum tractography for a 50-year-old healthy male subject.

**Figure 6:**
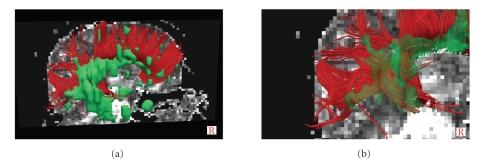
(a) Corpus callosum tractography for a 48-year-old relapsing remitting multiple sclerosis patient with lesional load of 16.4 mL. Lesions are superimposed on tractography and visualized with green blobs. (b) Zoom of posterior tracts which pass through the lesions of the same patient.

(i) Tractography algorithm was obtained with both the selected DTI sequences for all HV (in Figure 5 an example of CC tractography obtained with DTI-A is shown).

(ii) Tractography algorithm was obtained with both the selected DTI sequences for 28 of the 30 MS patients (Figure 6) but failed in two patients with a high number of lesions in CC.

## 4. Discussion

In this study we improved the quality of DTI sequences, looking for a compromise between SNR and spatial resolution. SNR values computed with different methods showed different bias and sensitivity to the noise level: this observation has to be further investigated. Despite that, at the aim of the present work, all methods were in accordance with the whole data set in pointing sequences DTI-A and DTI-B as the best ones without exception (SNR DTI-A > SNR DTI-B). These concordant evaluations allowed us to produce an automatic DTI sequences quality evaluation and to preliminary select two DTI sequences among 26. The two selected sequences had the best trade-off between SNR, voxel size, and diffusion sensing. Even if DTI-B has a lower SNR compared to DTI-A, the loss of maximum 15% in SNR was compensated by a higher resolution, which is a key element in determining tractographic reconstruction quality [7]. Both DTI sequences chosen through SNR-based evaluation are feasible for clinical protocols because of the acceptable acquisition time (about 3′). 

The optimum result is the production of CC individual-based tractography in 28 of 30 patients, with fiber tracts reconstructed even if they passed through a lesion. Both focal and diffuse alterations of tissue organization, which result in a decreased anisotropy and a consequent increase in uncertainty of the primary eigenvector of the DTI, are the well-known cause of the failure of tractography in MS in the previous studies [2, 28]. As previously described [7], the number of fibers decreases and tractography stops erroneously when SNR decreases. The improvement of SNR contributed on making possible the fiber bundles reconstruction. The high SNR is also fundamental for a better evaluation of MD and FA. Indeed, both of them are underestimated when SNR is low [29]. 

In order to increase SNR, more than one average is usually acquired, but too many averages amplify coregistration errors and raise acquisition time and subject movements. In our DTI protocol we choose to acquire 2 averages (runs) for every diffusion sequence. In Figure 1(b) the image is shown obtained by the difference between the first run and the second run (coregistered to the first one), which reveals that the ROI for the noise SD estimation (red) is put in a region with minimum error due to mismatch of coregistration: the difference image is uniform and does not have ringing or border artifacts.

**Figure 7:**
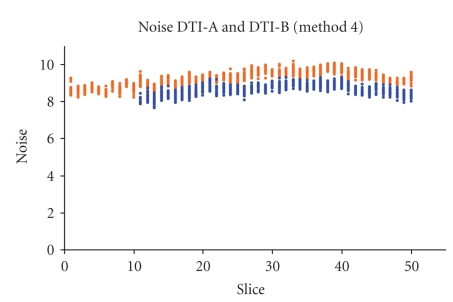
Comparison of Noise computed with method 4 for the two repetitions of 12 DTI gradient directions (*b*-value = 900 s/mm^2^, 50 slices) of DTI-A (orange dots) and the two repetitions of 12 DTI gradient directions (*b*-value = 1000 s/mm^2^, 40 slices) of DTI-B (blue dots).

The noise, estimated with different methods, is almost constant over the slices (Figure 7): for example, the DTI-A noise computed with method 4 has mean value (range) = 9.2 (8.2–10.2) over an image with mean (range) intensity of 33.7 (0–585); DTI-B noise computed with method 4 has mean value (range) = 8.6 (7.7–9.3) over an image with mean (range) intensity of 40.8 (0–681). Therefore, the SNR slices dependency (Figures 3 and 4) is mainly due to the mean signal differences for the various tissues acquired slice by slice.

Besides SNR examinations, even resolution has to be considered in DTI sequence parameters selection. Indeed, FA and MD are also influenced by the voxel size, due to the increment of the radial eigenvalues in a large voxel [30]. Furthermore, tissue with different diffusion properties can be inside a large voxel, bringing biased diffusion results [29]. This problem is known as partial volume effect and it causes an altered evaluation of DTI-derived measures, with a higher influence on FA than MD, due to the increase of the radial eigenvalues in a large voxel [30]. It is also known that the presence of crossing fibers within a large voxel influences the estimation of diffusion properties, since the apparent principal DT eigenvector is obtained as an average of the two crossing fibers' directions with a consequent reduction in the FA [7, 30]. 

For the above reasons we included also DTI-B in the clinical protocol, due to the smaller voxel size, even if DTI-A had the higher SNR.

Accurate FA and MD estimations improve the reliability of tractography, which is prone to errors: some of them are subjective (e.g., how the ROI for tracking selection is drawn, etc.) and some are intrinsic in the DTI sequence used. Indeed, bias in the estimation of diffusion tensor eigenvectors and eigenvalues damaged fiber tracking because it causes false or missing fibers [28, 30]. Several studies have been performed to reduce the errors on fiber tracking [30–33], but these methodologies are still being developed, none are used routinely, and most of them are time consuming and require strong computational power.

## 5. Conclusion

The results about SNR computed with different methods (Figures 3 and 4) showed that even those methods applied only on phantoms in previous studies [17, 21], or on mouse brain [12] or human abdomen [20] conventional MRI, can be successfully used also for DTI on human brain.

Both our selected DTI sequences were able to quantify a tissue damage in MS, leading to distinguish between MS patients and HV and between the different MS phenotypes. However, the sequence with higher resolution and higher *b*-value (DTI-B) achieved a better correlation with the presence of MS disease. Even if DTI-B sequence has less slices than DTI-A, it covered the entire CC tracts due to the acquired slab position. Appropriate positioning of the acquisition slab should be evaluated in further studies in order to analyze other fiber bundles. 

Finally, the proposed sequence and procedure showed higher reliability for fiber tracking and were able to discriminate the presence of MS disease even when severe lesional patterns were observed and may therefore be considered a potential powerful tool for studies to monitor the disease course and severity.

## Figures and Tables

**Table 1:** Intraclass correlation coefficient between measures derived from DTI-A and DTI-B.

DTI-derived metric	Intraclass correlation coefficient
GMMD	0.95
NAWMMD	0.99
NAWMFA	0.91

**Table 2:** Spearman's Correlation Coefficient between DTI-derived measures and the subjects' condition.

DTI-derived metric	Spearman's Correlation Coefficient	
	DTI-A	DTI-B
GMMD	0.57	0.68
NAWMMD	0.47	0.64
NAWMFA	−0.60	−0.70
CC-MD	0.63	0.78
CC-FA	−0.80	−0.84

## References

[1] Basser PJ, Mattiello J, Lebihan D (1994). Estimation of the effective self-diffusion tensor from the NMR spin echo. *Journal of Magnetic Resonance B*.

[2] Rovaris M, Agosta F, Pagani E, Filippi M (2009). Diffusion tensor MR imaging. *Neuroimaging Clinics of North America*.

[3] Cercignani M, Lannucci G, Filippi M (1999). Diffusion-weighted imaging in multiple sclerosis. *The Italian Journal of Neurological Sciences*.

[4] Miller DH, Thompson AJ, Filippi M (2003). Magnetic resonance studies of abnormalities in the normal appearing white matter and grey matter in multiple sclerosis. *Journal of Neurology*.

[5] Rovaris M, Gass A, Bammer R (2005). Diffusion MRI in multiple sclerosis. *Neurology*.

[6] Jones DK, Horsfield MA, Simmons A (1999). Optimal strategies for measuring diffusion in anisotropic systems by magnetic resonance imaging. *Magnetic Resonance in Medicine*.

[7] Liu X, Zhu T, Gu T, Zhong J Optimization of in vivo high-resolution DTI of non-human primates on a 3T human scanner.

[8] Henkelman RM (1985). Measurement of signal intensities in the presence of noise in MR images. *Medical Physics*.

[9] Kaufman L, Kramer DM, Crooks LE, Ortendahl DA (1989). Measuring signal-to-noise ratios in MR imaging. *Radiology*.

[10] Sano RM (1988). *MRI: Acceptance Testing and Quality Control: The Role of the Clinical Medical Physicist*.

[11] Murphy BW, Carson PL, Ellis JH, Zhang YT, Hyde RJ, Chenevert TL (1993). Signal-to-noise measures for magnetic resonance imagers. *Magnetic Resonance Imaging*.

[12] Sijbers J, den Dekker AJ, Poot D Robust estimation of the noise variance from background MR data.

[13] Sijbers J, den Dekker AJ, Van Audekerke J, Verhoye M, Van Dyck D (1998). Estimation of the noise in magnitude MR images. *Magnetic Resonance Imaging*.

[14] Mori S, Crain BJ, Chacko VP, van Zijl PCM (1999). Three-dimensional tracking of axonal projections in the brain by magnetic resonance imaging. *Annals of Neurology*.

[15] Conturo TE, Lori NF, Cull TS (1999). Tracking neuronal fiber pathways in the living human brain. *Proceedings of the National Academy of Sciences of the United States of America*.

[16] Basser PJ, Pajevic S, Pierpaoli C, Duda J, Aldroubi A (2000). In vivo fiber tractography using DT-MRI data. *Magnetic Resonance in Medicine*.

[17] Ogura A, Miyai A, Maeda F, Fukutake H, Kikumoto R (2003). Accuracy of signal-to-noise ratio measurement method for magnetic resonance images. *Japanese Journal of Radiological Technology*.

[18] National Electrical Manufacturers Association (NEMA) (2001). *Determination of Signal-to-Noise Ratio (SNR) in Diagnostic Magnetic Resonance Imaging*.

[19] Reeder SB, Wintersperger BJ, Dietrich O (2005). Practical approaches to the evaluation of signal-to-noise ratio performance with parallel imaging: application with cardiac imaging and a 32-channel cardiac coil. *Magnetic Resonance in Medicine*.

[20] Dietrich O, Raya JG, Reeder SB, Reiser MF, Schoenberg SO (2007). Measurement of signal-to-noise ratios in MR images: influence of multichannel coils, parallel imaging, and reconstruction filters. *Journal of Magnetic Resonance Imaging*.

[21] Imai H, Miyati T, Ogura A (2008). Signal-to-noise ratio measurement in parallel MRI with subtraction mapping and consecutive methods. *Nippon Hoshasen Gijutsu Gakkai Zasshi*.

[22] Weber D (1998). Quality Control Issues in MRI.

[23] Sijbers J, Poot D, den Dekker AJ, Pintjens W (2007). Automatic estimation of the noise variance from the histogram of a magnetic resonance image. *Physics in Medicine and Biology*.

[24] Ashburner J, Friston K (1997). Multimodal image coregistration and partitioning—a unified framework. *NeuroImage*.

[25] Basser PJ, Mattiello J, LeBihan D (1994). MR diffusion tensor spectroscopy and imaging. *Biophysical Journal*.

[26] Catani M, Thiebaut de Schotten M (2008). A diffusion tensor imaging tractography atlas for virtual in vivo dissections. *Cortex*.

[27] Pagani E, Rovaris M, Hirsch JG (2008). Optimising diffusion measurements for large-scale, multicentre multiple sclerosis trials: a pan-European study. *Journal of Neurology*.

[28] Ciccarelli O, Catani M, Johansen-Berg H, Clark C, Thompson A (2008). Diffusion-based tractography in neurological disorders: concepts, applications, and future developments. *The Lancet Neurology*.

[29] Jones DK, Basser PJ (2004). “Squashing peanuts and smashing pumpkins”: how noise distorts diffusion-weighted MR data. *Magnetic Resonance in Medicine*.

[30] Assaf Y, Pasternak O (2008). Diffusion tensor imaging (DTI)-based white matter mapping in brain research: a review. *Journal of Molecular Neuroscience*.

[31] Tuch DS, Reese TG, Wiegell MR, Makris N, Belliveau JW, Wedeen VJ (2002). High angular resolution diffusion imaging reveals intravoxel white matter fiber heterogeneity. *Magnetic Resonance in Medicine*.

[32] Tuch DS, Reese TG, Wiegell MR, Wedeen VJ (2003). Diffusion MRI of complex neural architecture. *Neuron*.

[33] Jansons KM, Alexander DC (2003). Persistent Angular Structure: new insights from diffusion MRI data. Dummy version. *Information Processing in Medical Imaging*.

